# Validation of CRP as prognostic marker for renal cell carcinoma in a large series of patients

**DOI:** 10.1186/1471-2407-12-399

**Published:** 2012-09-08

**Authors:** Sandra Steffens, Astrid Köhler, Raphael Rudolph, Hendrik Eggers, Christoph Seidel, Martin Janssen, Gerd Wegener, Mark Schrader, Markus A Kuczyk, Andres J Schrader

**Affiliations:** 1 Department of Urology, Hannover Medical School, Carl-Neuberg-Str. 1, D-30625, Hannover, Germany; 2 Department of Urology, Ulm Medical School, Hannover, Germany; 3 Department of Oncology, Hannover Medical School, Hannover, Germany; 4 Department of Urology, University Clinic of Saarland, Homburg, Germany; 5 Cancer Center, Hannover Medical School, Hannover, Germany

**Keywords:** Renal cell cancer, Biomarker, C-reactive protein, Prognosis, Survival

## Abstract

**Background:**

To evaluate the prognostic significance of the pre-operative C-reactive protein (CRP) serum level in patients with renal cell cancer (RCC).

**Methods:**

We evaluated 1,161 RCC patients with complete patient and tumour specific characteristics as well as information about their pre-operative CRP-level, who had undergone either radical nephrectomy or nephron-sparing surgery at two German high-volume centres (University Hospitals of Hannover and Ulm). The mean follow-up was 54 months.

**Results:**

The CRP-level, stratified to three subgroups (CRP ≤ 4, 4–10, and >10 mg/l), correlated significantly with tumour stage (p < 0.001), the risk of presenting nodal disease (2.1, 3.1, and 16.4%) and distant metastasis (2.9, 8.6, and 30.0%; p < 0.001). The Kaplan-Meier 5-year cancer specific survival (CSS) rates were 89.4, 77.9, and 49.5%, respectively (p < 0.001). Multivariate analysis identified CRP as an independent prognosticator for CSS as well as overall survival (p < 0.001). Patients with a CRP of 4–10 and >10 mg/l had a 1.67 and 2.48 fold higher risk of dying due to their RCC compared to those with a pre-operative CRP ≤4 mg/l, respectively.

**Conclusions:**

A high preoperative serum CRP level is an independent predictor of poor survival in patients with RCC. Its routine use could allow better risk stratification and risk-adjusted follow-up of RCC patients.

## Background

More than 40,000 new cases of renal cell carcinoma (RCC) are diagnosed in the European Union every year and about half of these patients will eventually die from RCC [1]. Despite increased health care facilities for imaging and consequent early diagnosis, still up to one third of all patients with RCC will have metastases at time of presentation [2]. Of the remaining two thirds, approximately 20–40% of those treated with (partial) nephrectomy in case of localized disease, do eventually develop metachronous metastasis or locally recurring cancer [3-5]. As patients’ clinical courses vary and are difficult to predict, the stratifications of patients to appropriate postoperative surveillance programs and different therapeutic strategies tailored to the risk of cancer progression will become increasingly important. Therefore, the area of defining new prognostic markers is of active interest [6,7], especially biomarkers in body fluids offer the opportunity for more objective and reproducible measurement prior to RCC surgery.

C-reactive protein (CRP) is an acute phase protein produced almost exclusively by the liver. CRP plasma levels can increase up to 1000-fold in response to microbial infection, trauma, infarction, autoimmune, or malignant diseases [8-11].

Elevated CRP levels can be a result of an underlying cancer and a premalignant state, respectively, as well as due to tumour growth associated tissue inflammation. However, it is still unclear if the tumor promotes inflammation or if inflammation promotes tumor aggressiveness. Experimental studies showed that at least some renal tumours produce interleukin-6, which promotes growth of RCC and therefore the presence of systemic inflammatory response could promote tumor aggressiveness [12,13].

A study published in 2009 by Allin et al. involving 10,408 individuals showed that elevated CRP is associated with increased risk of cancer, e.g. lung or colorectal malignancies [14]. Furthermore, an elevated CRP level was associated with an early death, even in patients without metastases [14]. Trichopoulos et al. [15] revealed that elevated CRP can be related to a higher risk of developing bladder cancer. Moreover, in patients with advanced bladder cancer undergoing chemotherapy elevated CRP levels were shown to be associated with a poor clinical outcome [16]. In addition, also in patients undergoing surgery for upper-tract urothelial carcinoma an increased CRP level seems to predict a poor survival [17]. Furthermore, other recent studies indicated that CRP, next to prostate specific antigen (PSA), could serve as an additional independent prognostic marker for tumor-specific survival in metastatic castration-resistant prostate cancer [18].

Smaller studies published in recent years which included from 40 up to 313 patients indicated that the preoperative CRP level could also be associated with RCC-specific mortality [19-26]. These smaller trials have shown a potential relationship between circulating CRP levels and tumour stage as well as a significant impact on the prognosis of patients with RCC.

In this large study including 1,116 patients, we have comprehensively analysed the potential pre-operative prognostic significance of CRP in patients with all stages and histological subtypes of RCC undergoing RCC-surgery.

## Patients and methods

### Patients and tumour characteristics

This study included 1,161 patients with complete patient and tumour specific characteristics as well as information about their pre-operative CRP-level, who underwent renal tumour surgery 1990–2010 for RCC at the Hannover (1995–2006) or Ulm (1995–2010) University Medical Centres. The histological tumour subtype was determined according to the 1997 UICC Classification. Staging was based on the 2002 TNM Classification. Information on patients’ and tumour characteristics, such as age, sex, stage, presence of regional lymph node or distant metastases, histological subtype, Fuhrman grade, and CRP-value, was obtained from our computerized institutional databases.

The pre-operative CRP-value was categorized according to Johnson et al. [27], i.e. low level: CRP ≤ 4 mg/l, intermediate: 4–10 mg/l, and high: >10 mg/l.

### Follow up

The duration of the follow-up was calculated from date of surgery to the date of death or last follow-up. Death was assessed as either cancer-related or -unrelated. The primary end point of this study was cancer-specific survival (CSS). Information about the exact date as well as cause of death for each patient was received from the patient’s general practitioner, a close family member or the patient’s hospital records if she/he had been followed up or died in one of our institutions. Follow-up assessment ended in October 2011. Until then, all patients’ data were updated at least every 6 months on a regular basis.

### Statistical methods

Continuous variables were reported as mean value and standard deviation (SD) or median value and interquartile ranges (IQR) in the case of parametric or non-parametric distribution, respectively. Chi^2^ tests were conducted to assess correlations of covariate distributions and CRP-groups.

Receiver operation characteristics (ROC) curves were constructed to assess the potential of preoperative CRP to predict overall and cancer-specific survival and to identify cut-offs to categorise CRP-levels in risk groups.

Kaplan-Meier estimates of survival time were calculated, and subgroups were compared by the log rank test statistic. Multivariate Cox regression models were used to assess the association between survival and CRP-levels adjusted for different clinical and patient covariates (i.e., age, sex, tumour stage and grade, the histological subtype, and metastatic status). SPSS 19.0 was used for statistical assessment. In all tests, a two-sided p < 0.05 was considered to indicate significance.

## Results

Our patient population of 761 (65.6%) men and 400 (34.4%) women had a mean age of 61.9 years (19–90). 989 (87.1%), 86 (7.6%), 26 (2.3%), and 34 (3.0%) of all patients suffered from clear cell, papillary, chromophobe, and non-classified RCC, respectively. The median body mass index (BMI) for all patients was 26.5 kg/m^2^ (IQR, 24.0 – 29.4). 880 (75.9%) and 281 (24.1%) were treated with radical and partial nephrectomy. Detailed patients’ and tumour characteristics including stage and grade are summarized in Table 1.

**Table 1:** Association between different patient and cancer-specific variables with the pre-operative CRP value

**Variable**	**CRP <4 mg/l**	**CRP 4–10 mg/l**	**CRP >10 mg/l**	**p-value**	**Test**
Age, mean (95% CI)	60.9 (59.9 - 61.9)	62.1 (60.4 - 63.8)	63.4 (62.4 - 64.5)	0.005	ANOVA
Sex				0.28	chi-square
female	225 (36.5%)	51 (31.3%)	124 (32.5%)		
male	391 (63.5%)	112 (68.7%)	258 (67.5)		
Side				0.81	chi-square
right	325 (52.8%)	86 (52.8%)	193 (50.5%)		
left	286 (46.5%)	77 (47.2%)	187 (49.0%)		
bilateral	4 (0.7%)	0	2 (0.5%)		
Type of surgery				<0.001	chi-square
Radical nephrectomy	408 (66.2%)	130 (79.8%)	342 (89.8%)		
Partial nephrectomy	208 (33.8%)	33 (20.2%)	39 (10.2%)		
Stage				<0.001	chi-square
pT1a	284 (46.6%)	58 (35.8%)	39 (10.4%)		
pT1b	164 (26.9%)	40 (24.7%)	55 (14.7%)		
pT2	44 (7.2%)	15 (9.3%)	44 (11.7%)		
pT3a	66 (10.8%)	23 (14.2%)	78 (20.8%)		
pT3b/c	49 (8.2%)	26 (16.0%)	142 (37.9%)		
pT4	2 (0.3%)	0	17 (4.5%)		
LN metastasis^1^	13 (2.1%)	5 (3.1%)	62 (16.4%)	<0.001	chi-square
Pulmonal/visceral metastasis^1^	18 (2.9%)	14 (8.6%)	114 (30.0%)	<0.001	chi-square
Grade				<0.001	chi-square
G1	144 (23.8%)	30 (18.9%)	26 (7.0%)		
G2	416 (68.8%)	110 (69.2%)	212 (56.7%)		
G3	45 (7.4%)	18 (11.3%)	127 (34.0%)		
G4	0	1 (0.6%)	9 (2.4%)		
Histological subtype				0.08	chi-square
non ccRCC	86 (14.3%)	24 (14.9%)	36 (9.7%)		
ccRCC	516 (85.7%)	137 (85.1%)	336 (90.2%)		

^1^at time of renal surgery. Abbreviations: *CRP* = C reactive protein, *RCC* = renal cell carcinoma, *ccRCC* = clear-cell RCC*, LN* = lymph node.

The median/mean follow-up was 46/54 months (IQR, 19 – 84). By the last day of data acquisition, 279 (24.0%) had died from their tumour disease and 48 (4.1%) from other causes.

### Clinical parameters

Mean (median) pre-operative CRP value was 21.6 (4.0) mg/l. The mean (median) CRP value in the three subgroups (CRP ≤ 4, 4–10, and >10 mg/l) was 2.5 (2.6), 6.9 (6.8), and 58.6 (38.0) mg/l, respectively. The three groups were comparable concerning the distribution of sexes (p = 0.28; Chi^2^-Test, Table 1). However, patients with a higher CRP level were significantly older (mean, 60.9 vs. 62.1 vs. 63.4 years; p = 0.005; ANOVA). Moreover, patients with a BMI <25 had significantly more often high CRP values than those with a BMI >30 kg/m^2^ (35.8 vs. 24.7%; p < 0.001; Chi^2^ test).

### Tumour-specific parameters

The CRP-level correlated significantly with the tumour stage: 19.3, 30.2, and 63.2% of all patients with a CRP ≤4, 4–10, and >10 mg/l suffered from locally advanced (pT ≥ 3, N_any_, M_any_) RCC at the time diagnosis (p < 0.001, Chi^2^ test). The risk of presenting nodal disease (2.1, 3.1, and 16.4%) or distant metastasis (2.9, 8.6, and 30.0%) also increased significantly in each CRP-group (p < 0.001, Chi^2^ test; Table 1). Accordingly, the median CRP value was significantly higher in advanced (pT3-4 and/or N/M+) than in localized (pT1-2, N/M-) disease (16.0 vs. 4.0 mg/l; p < 0.001, Mann–Whitney-test).

Moreover, high (>10 mg/dl) CRP-levels were found in 13.0, 28.7, 66.8, and 90.0% of patients with G1, G2, G3, and G4 differentiated RCC (p < 0.001, Chi^2^ test).

### Clinical course and oncological outcome

**Figure 1:**
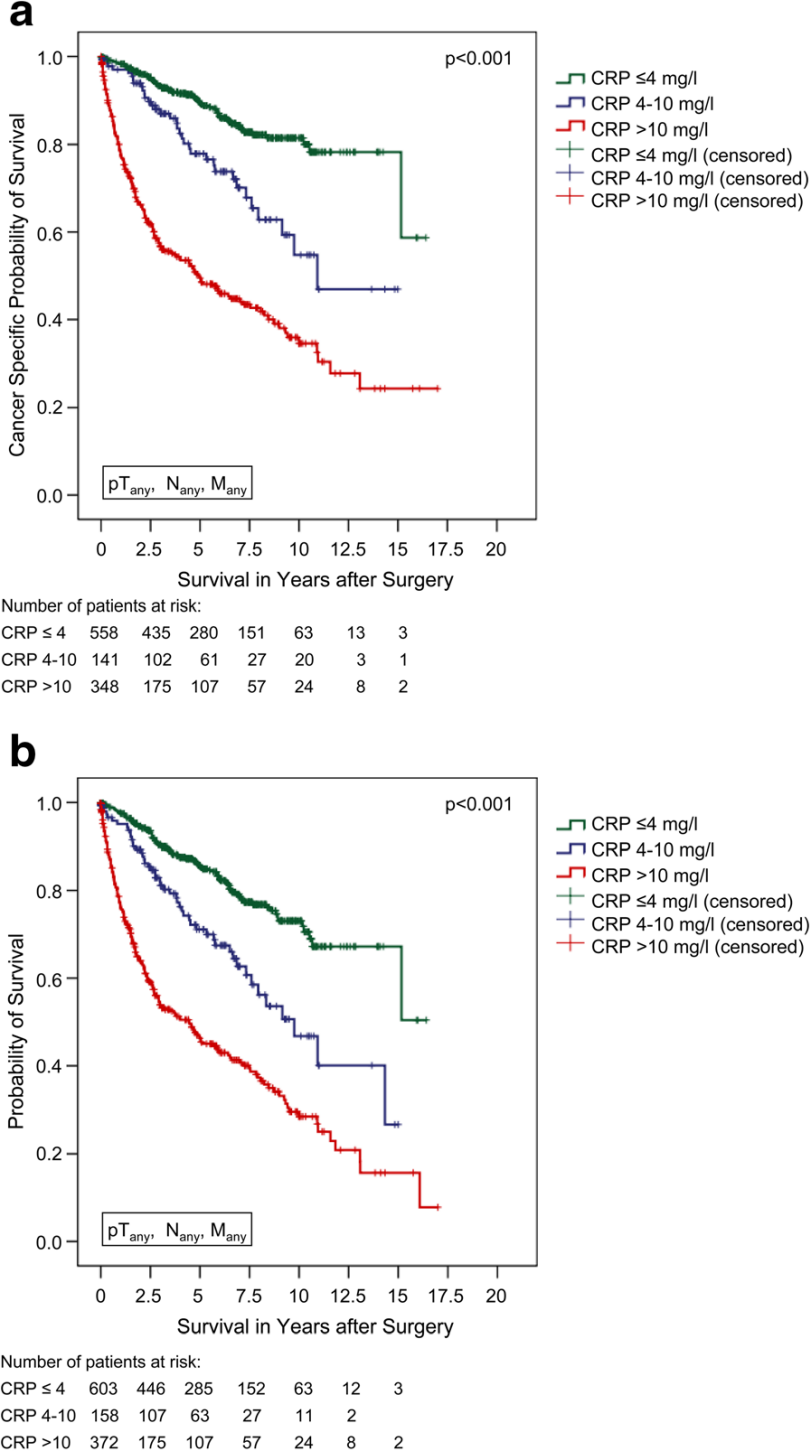
**a) Cancer-specific survival (Kaplan-Meier) for all RCC patients plotted against the pre-operative CRP-group.** The 5-year survival rate was 89.4%, 77.9%, and 49.5% for all evaluable patients (n = 1,047) with a CRP of ≤4 mg/l (n = 558), 4–10 mg/l (n = 141), and >10 mg/l (n = 348), respectively (p < 0.001, log rank). **b**) Overall survival (Kaplan-Meier) for all RCC patients plotted against the pre-operative CRP-group. The 5-year survival rate was 85.5%, 71.1%, and 46.3% for all evaluable patients (n = 1,133) with a CRP of ≤4 mg/l (n = 603), 4–10 mg/l (n = 158), and >10 mg/l (n = 372), respectively (p < 0.001, log rank).

After a mean follow-up of more than 4 years, the tumour-associated death rates were 12.4, 24.8, and 50.7% in the CRP ≤4, 4–10, and >10 mg/l groups (p < 0.001, Chi^2^ test). In accordance, the Kaplan-Meier 5-year CSS rates were 89.4, 77.9, and 49.5%, respectively (p < 0.001, log rank; figure 1a). The Kaplan-Meier 5-year overall survival rates were calculated at 85.5, 71.1, and 46.3%, respectively (p < 0.001, log rank; figure 1b).

Applying receiver-operating characteristic analyses, the CRP value exhibited an AUC (95% CI) of 0.78 (0.75 – 0.81; p < 0.001) for cancer specific and of 0.74 (0.71-0.78; p < 0.001) for overall survival. Moreover, the cut-off values 4.0 and 10.0 mg/l, as suggested earlier in a smaller trial [27] could be confirmed as highly discriminating concerning risk classification.

**Figure 2:**
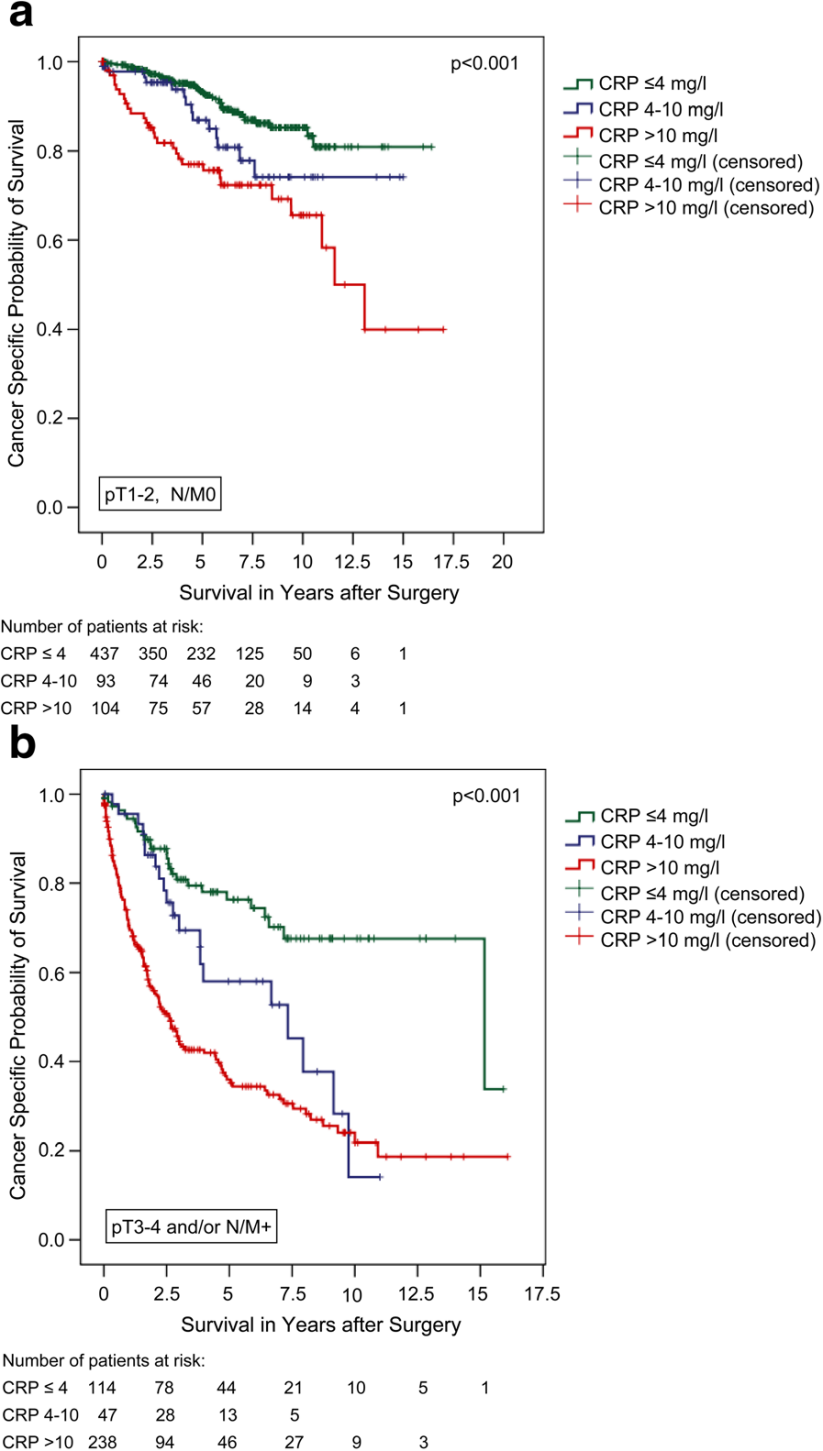
**a) Cancer-specific survival (Kaplan-Meier) for organ-confined RCC plotted against the pre-operative CRP-group:** The 5-year survival rate was 93.2%, 86.9%, and 77.0% for all evaluable patients (n = 634) with a CRP of ≤4 mg/l (n = 437), 4–10 mg/l (n = 93), and >10 mg/l (n = 104), respectively (p < 0.001, log rank). **b**) Cancer-specific survival (Kaplan-Meier) for advanced RCC plotted against the pre-operative CRP-group: The 5-year survival rate was 76.3%, 58.0%, and 35.9% for all evaluable patients (n = 399) with a CRP of ≤4 mg/l (n = 114), 4–10 mg/l (n = 47), and >10 mg/l (n = 238), respectively (p < 0.001, log rank).

Subgroup analyses comparing localized (pT1-2, N/M0) and advanced disease (pT3-4 and/or N/M+) confirmed significant differences in CSS for all three CRP-groups in both clinical settings (figures 2a + b). The Kaplan-Meier 5-year CSS rates were 93.2, 86.9, and 77.0% for localized (p < 0.001; log rank) and 76.3, 58.0, and 35.9% for advanced RCC, respectively (p < 0.001; log rank).

Multivariate analysis including age, sex, histological subtype, tumour stage, and differentiation identified the CRP-value as a continuous metric variable as an independent prognosticator for cancer-specific (HR 1.007, 95% CI: 1.004-1.009; p < 0.001, Cox regression) as well as overall survival (HR 1.006, 95% CI: 1.004-1.008; p < 0.001, Cox regression) in patients with RCC. After its stratification into the three groups (CRP ≤4, 4–10, and >10 mg/l) the discriminative power and prognostic significance of the CRP value became even more evident. Patients with a CRP of 4–10 and >10 mg/l had a 1.67 and 2.48 fold higher risk of dying due to their RCC compared to those with a pre-operative CRP ≤4 mg/l, respectively (Table 2).

**Table 2:** Multivariable analysis revealed that the pre-operative CRP-level is an independent prognostic marker for cancer- specific survival

**Variable**	**P value**	**HR (95%CI)**
Age [years] ^1^	<0.001	1.03 (1.01-1.04)
Sex	0.74	
female		1
male		0.96 (0.72-1.26)
T stage	0.07	
pT1a		1
pT1b	0.74	1.08 (0.69-1.69)
pT2	0.34	1.30 (0.76-2.22)
pT3a	0.006	1.90 (1.20-2.99)
pT3b	0.20	1.35 (0.86-2.13)
pT3c	0.35	2.00 (0.46-8.59)
pT4	0.89	0.94 (0.42-2.20)
LN metastases ^2^	<0.001	
N-		1
N+		3.59 (2.46-5.25)
Pulmonary or visceral metastases ^2^	<0.001	
M-		1
M+		3.23 (2.31-4.50)
Differentiation	0.02	
G1		1
G2	0.01	1.97 (1.16-3.34)
G3	0.003	2.47 (1.36-4.51)
G4	0.01	4.13 (1.38-12.34)
Histological subtype	0.17	
non ccRCC		1
ccRCC		1.43 (0.85-2.41)
CRP-value ^2^	<0.001	
<4 mg/l		1
4-10 mg/l	0.03	1.66 (1.06-2.59)
>10 mg/l	<0.001	2.58 (1.83-3.64)

^1^continuous metric variable, ^2^ at the time of surgery.

## Discussion and conclusions

In this large study we could reveal that different CRP leves were significantly associated with tumour stage, grade, and a poor cancer specific and overall survival in patients undergoing resection for RCC. These results confirmed the association of circulating CRP levels with the tumour stage and its impact on the prognosis of patients with RCC. Previous smaller studies which had included up to 313 patients [24], only, already indicated that CRP might be an independent predictor of cancer specific survival in patients with RCC.

Only recently, Johnson et al. [27] presented a study suggesting a CRP-based prognostic classification of patients with localized RCC. The authors recommended to stratify patients according to the CRP cut-off values 4.0 and 10.0 mg/l. We were able to confirm these cut-off values as highly discriminating concerning both overall and also cancer specific survival. Furthermore, with our larger patient cohort, compared to the study by Johnson et al. [27] (n = 1,161 vs. n = 173), we were able to prove the impact of the CRP-level as an independent prognostic predictor for both patients with localized and advanced disease.

Lamb et al. [25] indicated that the presence of a preoperative systemic inflammatory response measured in an elevated CRP level might be an independent negative predictor for relapse-free survival in patients with localized RCC after curative surgery. However, Lamb and co-workers evaluated 60 patients with localized clear cell RCC, only, and used a CRP level of >10 mg/l as cut off point.

An elevated circulating CRP concentration had also been suggested to be a poor prognostic factor in patients with metastatic RCC [10,12,28]. Masuda et al. [29] published a retrospective study including patients with advanced RCC which identified the CRP-level as a prognostic factor independent of tumour stage and grade. However, the threshold for CRP was not defined in their survival analysis. In contrast, Ito et al. [22] were able to demonstrate in a cohort of 178 patients that a CRP elevation >10 mg/l might be an independent predictor for recurrence and prognosis in both localized and metastatic RCC. Interestingly, the mean CRP levels in the study by Ito et al. [22] and our own study were similar, i.e. 21 mg/l and 21.6 mg/l, respectively.

Lamb et al. [25] showed that the tumour cell expression of IL-6 was not significantly associated with circulating CRP levels hypothesizing that the main source of IL-6 causing an elevated CRP level is not the tumour itself. In contrast, in 2005 Jabs et al. [25] published a study with 40 patients suggesting that the CRP expression by the tumour itself is directly associated with the circulating CRP concentration indicating an autonomous production of CRP in the tumor and normal kidney tissue.

Accordingly, Johnson et al. [30] only recently evaluated the influence of intratumoral CRP on overall survival in 95 patients with localized clear cell RCC using immunohistochemical analysis. The tumours were categorized into low, intermediate, and high CRP staining intensity. Mean overall survival was significantly longer in the low (44.2 months) and intermediate (40.5 months) risk (i.e. CRP) group compared to the group of tumours expressing high amounts of CRP (31.6 months; p = 0.002 and p = 0.067). Applying multivariate analysis, patients with high intratumoural CRP levels experienced a 12-fold increased risk of overall mortality compared to patients with low CRP expressing tumours.

As biomarkers in fluids offer the opportunity for more objective and reproducible measurement prior to tumour surgery, the use of CRP as a well-standardized parameter worldwide, should not be underestimated. Rather than tumour tissue-based factors, it can easily be implemented as a prognostic factor in addition to tumour stage and grade, to more accurately stratify patients with RCC. Karakiewicz et al. [24] were able to show in a group of 313 patients that the incorporation of the CRP value into the UISS scoring systems for patients with localized RCC might improve its prognostic significance. Furthermore, Iimura et al. introduced the TNM-C Score, a prediction model including C-reactive protein in patients treated with nephrectomy for clear cell RCC [19]. In a cohort of 249 patients - and 290 for external validation - they were able to show that the model is a useful tool to predict cancer specific survival [19]. However, to our knowledge, the incorporation of CRP in a prognostic model is not yet an established tool used in clinical routine.

In conclusion, this large study confirms that the preoperative CRP-level is an independent prognostic factor in patients with RCC. A high preoperative serum CRP level is significantly associated with poor survival in patients with both localized and advanced RCC. Its routine use could allow better risk stratification and risk-adjusted follow-up for patients with kidney cancer.

## Competing interests

All authors declare that they have no competing interests.

## Authors’ contributions

SS designed and supervised the study, took part of the data acquisition and interpretation as well as drafting of the manuscript. AK carried out the data acquisition, participated in the data interpretation and drafting of the manuscript. RR, HE and GW carried out the data acquisition. CS, MJ, MS and MAK revised the manuscript for important intellectual content. AJS performed the statistical analysis and interpretation of the data, supervised the study and was involved in the drafting of the manuscript. All authors read and approved the final manuscript.

## Pre-publication history

The pre-publication history for this paper can be accessed here:

http://www.biomedcentral.com/1471-2407/12/399/prepub
